# Proteomic analysis by iTRAQ-PRM provides integrated insight into mechanisms of resistance in pepper to *Bemisia tabaci* (Gennadius)

**DOI:** 10.1186/s12870-019-1849-0

**Published:** 2019-06-21

**Authors:** Xiaoxia Wu, Jiaxing Yan, Yahong Wu, Haibo Zhang, Shuangrong Mo, Xiaoying Xu, Fucai Zhou, Haidong Ding

**Affiliations:** 1 Joint International Research Laboratory of Agriculture and Agri-Product Safety, College of Bioscience and Biotechnology, Yangzhou University, Yangzhou, 225009 China; 2 College of Horticulture and Plant Protection, Yangzhou University, Yangzhou, 225009 China

**Keywords:** Pepper, *Bemisia tabaci*, Resistance, Proteome, iTRAQ, PRM

## Abstract

**Background:**

The *Bemisia tabaci* is a major leaf feeding insect pest to pepper (*Capsicum annuum*), causing serious damage to pepper growth and yield. It is particularly important to study the mechanism of pepper resistance to *B. tabaci*, and to breed and promote the varieties of pepper resistant to *B. tabaci*. However, very limited molecular mechanism is available about how plants perceive and defend themselves from the destructive pest. Proteome technologies have provided an idea method for studying plant physiological processes in response to *B. tabaci*.

**Results:**

Here, a highly resistant genotype and a highly susceptible genotype were exposed to *B. tabaci* feeding for 48 h to explore the defense mechanisms of pepper resistance to *B. tabaci*. The proteomic differences between both genotypes were compared using isobaric tag for relative and absolute quantification (iTRAQ). The quantitative data were validated by parallel reaction monitoring (PRM). The results showed that 37 differential abundance proteins (DAPs) were identified in the RG (resistant genotype), while 17 DAPs were identified in the SG (susceptible genotype) at 48 h after *B. tabaci* feeding. 77 DAPs were identified when comparing RG with SG without feeding. The DAP functions were determined for the classification of the pathways, mainly involved in redox regulation, stress response, protein metabolism, lipid metabolism and carbon metabolism. Some candidate DAPs are closely related to *B. tabaci* resistance such as annexin D4-like (ANN4), calreticulin-3 (CRT3), heme-binding protein 2-like (HBP1), acidic endochitinase pcht28-like (PR3) and lipoxygenase 2 (LOX2).

**Conclusions:**

Taken together, this study indicates complex resistance-related events in *B. tabaci* interaction, provides novel insights into the molecular mechanism underlying the response of plant to *B. tabaci*, and identifies some candidate proteins against *B. tabaci* attack.

**Electronic supplementary material:**

The online version of this article (10.1186/s12870-019-1849-0) contains supplementary material, which is available to authorized users.

## Background

*Bemisia tabaci* (Gennadius) is one of the most widely distributed agricultural pests that may be harmful to many arable and horticultural crops such as *Solanaceae*, *Cucurbitaceae* and *Cruciferae*. Pepper (*Capsicum annuum*), an important vegetable type in China, is one of the most serious hosts of *B. tabaci*. *B. tabaci* complex contains more than 24 morphologically indistinguishable biotypes [1]. Phylogenetic studies based on mitochondrial cytochrome oxidase I (mtCOI) gene revealed that *B. tabaci* contains at least 34 putative species [2]. During the past two decades, *B. tabaci* biotype B has been introduced into at least 54 countries from its origin in the Middle East-Asia Minor region and become a world-wide invasive and destructive whitefly species. In China, *B. tabaci* was first recorded in the late 1940s, but was not recorded as the major agricultural pest until the introduction of *B. tabaci* biotype B in the mid-1990s [3].

*B. tabaci* not only damages plants by sucking vital sap from the phloem tissue but also causes indirect damage by vectoring many kinds of viruses and by promoting the growth of saprophytic fungi on the leaves [4, 5]. The visible, direct damage caused by *B. tabaci* are leaf deformation and honeydew secretion. The control of *B. tabaci* has been relied heavily on the synthetic insecticides, but the pesticide resistance has been developed in many parts of the world. For example, *B. tabaci* field populations were found highly resistant to imidacloprid and thiamethoxam, both in Israel, Spain, Crete, and China [3]. Plant–insect interactions have resulted in the evolution of sophisticated mechanisms that respond to insect attack [6]. A promising alternative to control *B. tabaci* is to study the resistant mechanism of host-plants, explore resistant genes, and breed for durable host-plant resistance [4, 7]. Therefore, it is particularly important to study the mechanism of pepper resistance to *B. tabaci*, and to breed and promote the varieties of pepper resistant to *B. tabaci*.

Similar to pathogen-plant interaction, *B. tabaci* induces salicylic acid (SA) defenses and suppresses effectual jasmonic acid (JA) defenses in Arabidopsis [8]. In tomato, *B. tabaci* causes JA levels increase initially and decline within days, whereas the expression of SA-regulated genes was gradually increased [9, 10] Feeding by *B. tabaci* is known to induce specific genes such as *WFI1* in tomato and *SLW1* in squash [11]. Tomato pathogenesis-related protein (PR) genes are also expressed in response to *B. tabaci* biotype B feeding [12]. Recently, RNA-Seq datasets analysis revealed a comprehensive insect resistance response mechanism in cotton to infestation by *B. tabaci* and showed that MPK-WRKY-JA and ethylene (ET) pathways might regulate cotton defenses to *B. tabaci* [13].

Despite these advances, the comprehensive molecular mechanisms underlying plant resistant to *B. tabaci* remain poorly defined. So far, most of these studies focus on nucleic acid level, but fewer have studied the actual protein. Proteome technologies provide an idea method for studying plant physiological processes. Recently, the proteome changes of *Arabidopsis thaliana* leaves infested by *B. tabaci* were reported using two-dimensional electrophoresis and mass spectrometry [6]. Ibrahim et al. [14] showed that the major proteins like MAP kinases, COBRA-like protein family and NBS disease resistance protein were expressed under infested conditions using one-dimensional electrophoresis following liquid chromatography coupled with tandem mass spectrometry (LC–MS/MS). To investigate the pepper-*B. tabaci* interaction, two genotypes were identified exhibiting different *B. tabaci* susceptibilities, one that was a highly resistant genotype (termed RG) and another that was a highly susceptible genotype (termed SG), and the proteomic differences between both genotypes after *B. tabaci* infestation for 48 h were compared using isobaric tag for relative and absolute quantification (iTRAQ). The results may contribute to our understanding of protein response and alteration and provide insights into the molecular mechanisms involved in response to *B. tabaci* infestation in plants.

## Results

### Identification of *B. tabaci* resistance in peppers

**Fig. 1:**
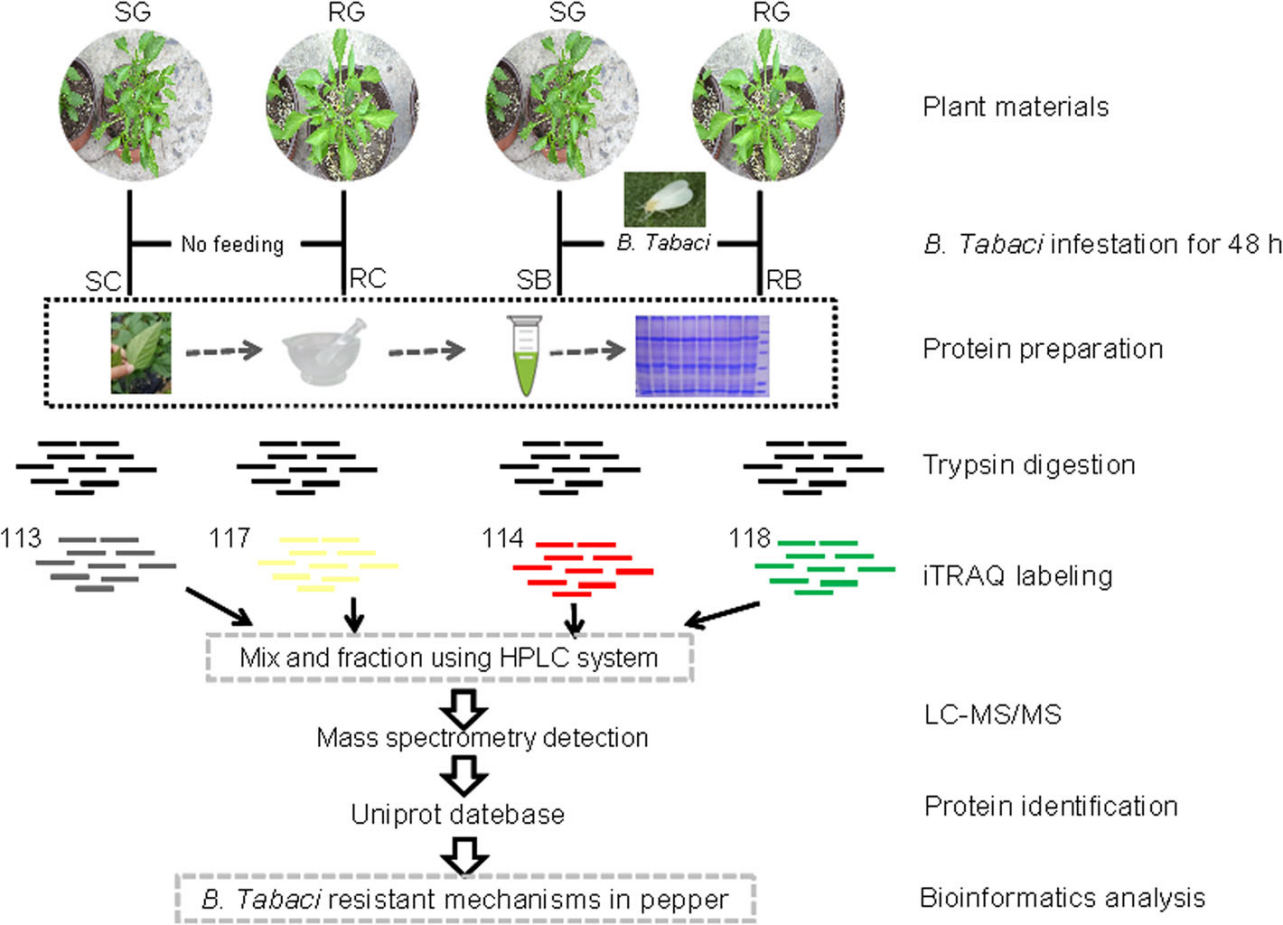
Strategy for analysis of protein expression in pepper leaves by 4-plex isobaric tagging. SG, susceptible genotype; RG, resistant genotype; SB, susceptible genotype infested with *B. tabaci*; RB, resistant genotype infested with *B. tabaci*; SC, SB control; RC, RB control

**Fig. 2:**
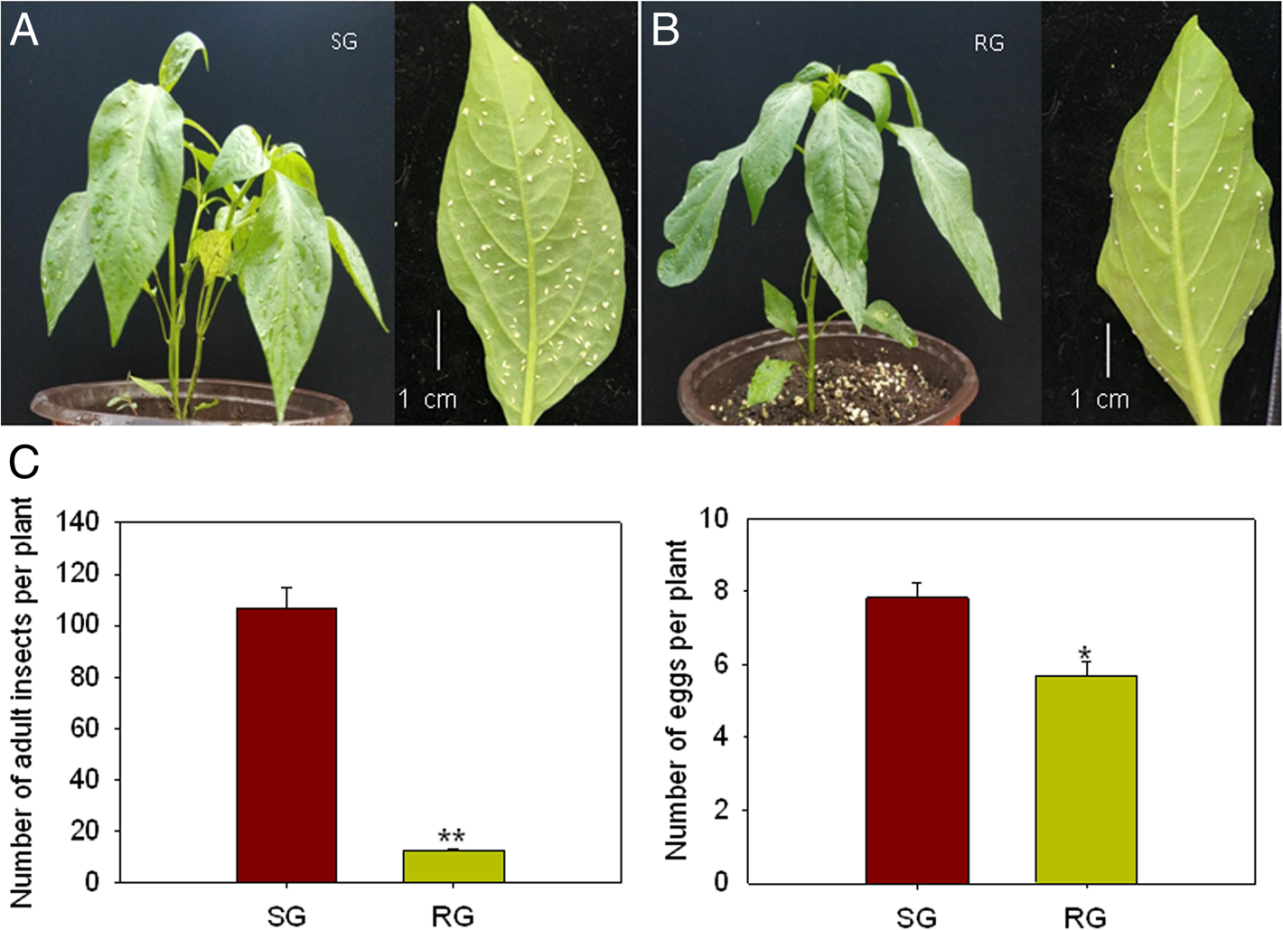
Symptom of different pepper genotypes, a susceptible genotype (SG) and a resistant genotype (RG) exposed to *B. tabaci* feeding. **a** Representative images of the SG following *B. tabaci* infestation. **b** Representative images of the RG following *B. tabaci* infestation. Left, the plant phenotype; Right, the mature *B. tabaci* populations on single leaf from either the SG or RG. Plants were infested with *B. tabaci* in the greenhouse for 72 h. **c** Anti-selectivity of different pepper varieties on *B. tabaci*

In the preliminary experiment, we screened a lot of pepper materials and two pepper genotype varieties were identified showing either high levels of resistance (RG) or susceptibility (SG) to *B. tabaci* infestation. The resistant characteristics of both genotypes were investigated after *B. tabaci* infestation (Figs. 1 and 2). It is observed that the leaf of RG showed deep green and the leaves of SG looks displayed light green color (Fig. 2 a, b). Besides, different settling behavior of *B. tabaci* adults was found between two varieties. The population of *B. tabaci* settled on SG was about 40 times higher than on RG. Similarly, higher egg hatchability was observed in SG whereas lower egg hatchability was observed in RG (Fig. 2c) Therefore, the two genotypes are ideal candidates for studying the proteomic mechanisms of pepper in response to *B. tabaci* infestation.

### iTRAQ protein profiling

To investigate the mechanisms of pepper resistance against *B. tabaci*, the proteomic profiling analysis at 48 h after *B. tabaci* treatment of resistant and susceptible genotypes was performed using iTRAQ. The average of the spectral identification rates of the secondary mass spectra produced by the three replicates reached 34% or more. For example, in the replicate one, a total of 397,554 spectra were obtained, in which 13,5885 spectra were matched to the known database and the spectral identification rate is 31.2%. A total of 20,102 peptides and 2756 proteins (at least two unique peptides with high confidence) were identified by iTRAQ analysis against the Uniprot database *Capsicum annuum* (39,809 items) (Additional file 4: Table S1).

**Fig. 3:**
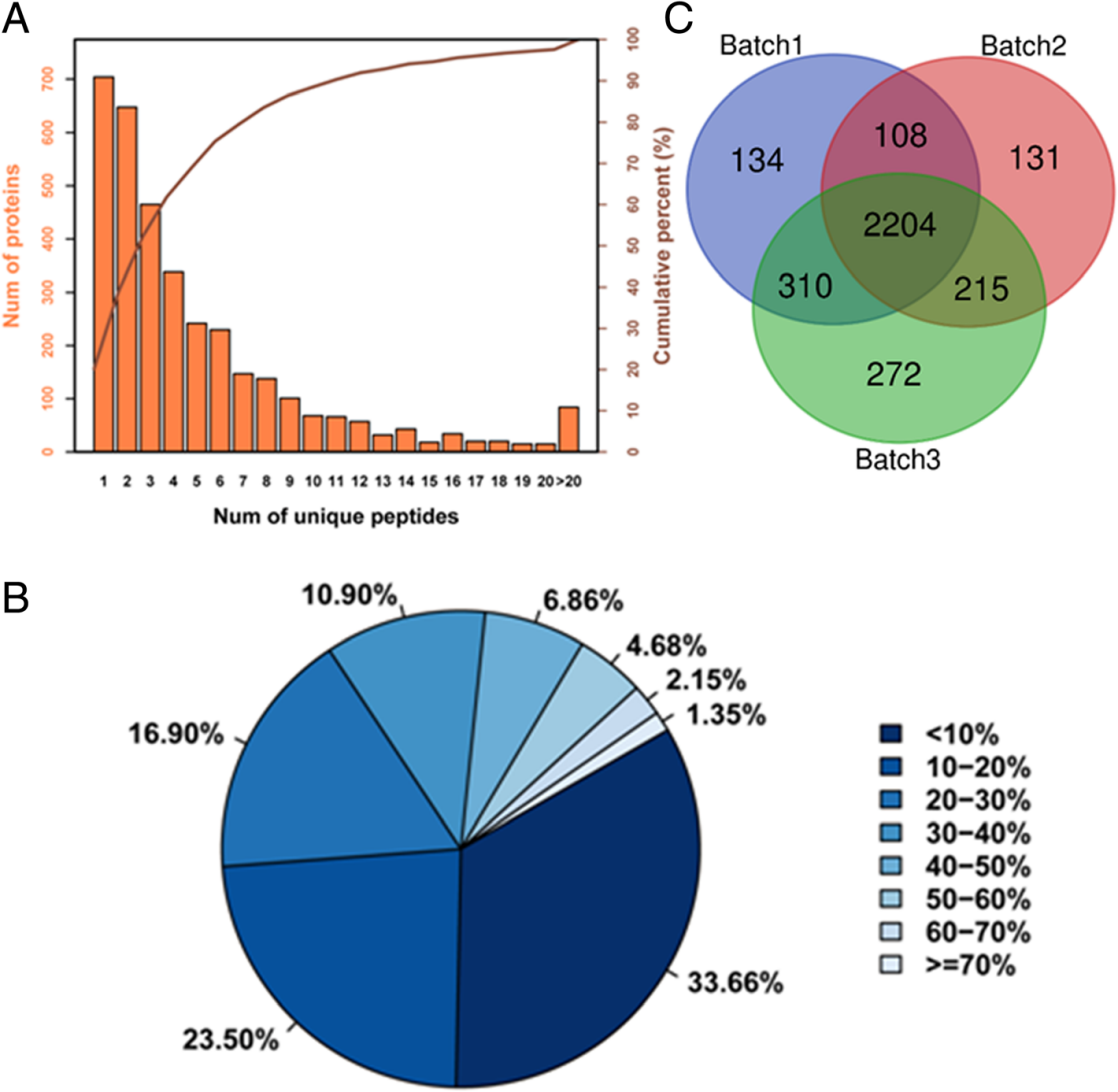
Identification and analysis of the proteome by iTRAQ **a** Distribution of the number of peptides. The X axis represents the scope of the number of unique peptides, and the Y axis represents the number of proteins and corresponding cumulative percent. **b** Distribution of the proteins’sequence coverage. The pie chart displays the proportion of the number of the different proteins within the scope of coverage in the total protein amount. **c** Venn diagram of unique and shared proteins of three batches

The peptide number analysis of the identified proteins showed that the peptide segment numbers in the most of proteins were identified to contain less than 12 (Fig. 3a). The number of proteins containing at least 2 unique peptides in the three batches of this study were 2756, 2658, 3001, accounting for 79.80, 79.56, and 80.65% of the total protein, respectively. The percentage of protein with a coverage of [0, 10%] is 33.66%, the protein with coverage greater than or equal to 20% accounted for 42.84% of the total protein, and the average of protein identification coverage was 21.07% (Fig. 3b). Besides, venn diagram of three batches showed that there were about 80% shared proteins indicating the high repeatability (Fig. 3c).

Among all three replicates, all proteins were annotated to 52 gene ontology (GO) terms by GO analysis (Additional file 5: Table S2). In terms of biological process categories, most proteins were categorized into the metabolic process (3193, 74.6%), cellular process (3166, 74.0%) and response to stimulus (1364, 31.9%). The major cellular components were cell (3692, 86.2%) and cell part (3687, 86.1%). The largest molecular functions of proteins obtained by GO analysis were catalytic activity (2118, 49.5%) and binding (1998, 46.7%). Using pathway analysis, 2884 proteins were annotated to 116 pathways including metabolic pathways, Biosynthesis of secondary metabolites, microbial metabolism in diverse environments, ribosome, spliceosome, plant-pathogen interaction, etc.

### Identification of DAPs after *B. tabaci* infestation

**Fig. 4:**
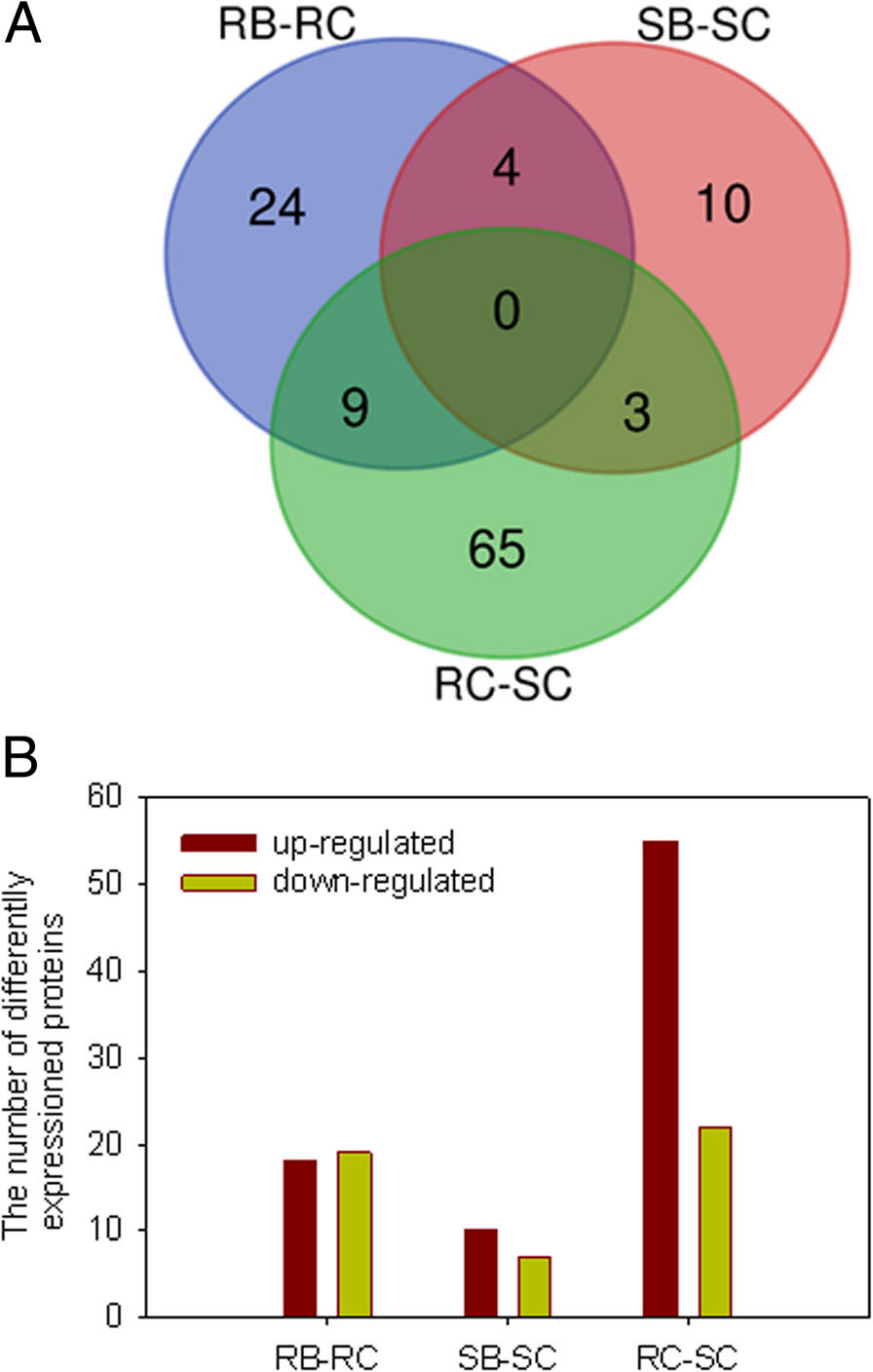
Venn diagram of differential abundance proteins (DAPs). **a** Venn diagram; **b** The number of up-regulated and down-regulated DAPs

To examine DAPs in response to *B. tabaci*, the proteome changes between two genotypes in response to *B. tabaci* challenge were investigated in three independent iTRAQ experiments. Compared to the control group, a 1.50-fold or 0.67-fold change threshold with a *P*-value < 0.05 in protein expression in at least two experiments were classified as a physiologically significant change. We analyzed DAPs between RG inoculated with *B. tabaci* (RB) and RG control (RC), between SG inoculated with *B. tabaci* (SB) and SG control (SC), and between RC and SC. A total of 115 DAPs was identified from “RB-RC”, “SB-SC” and “RC-SC”. A venn diagram including the total 115 proteins was generated (Fig. 4). The detailed information of all proteins obtained from three biological replicates is presented in Table 1. After *B. tabaci* infestation, 24 and 10 DAPs emerged differential accumulation in RB and SB, respectively, but these proteins have no difference in RC-SC. Of the 37 DAPs in RB, 18 proteins were up-regulated and 19 proteins were down-regulated. Among 17 DAPs of SB, 10 proteins were up-regulated and 7 proteins were down-regulated. However, 9 and 3 protein levels had changed in RC compared with SC (RC-SC)". These specific *B. tabaci* responsive proteins might be important factor for resistance to *B. tabaci*. In the venn diagram, 4 DAPs were shared between two genotypes after *B. tabaci* attack, including 60S ribosomal protein L4, xyloglucan endotransglucosylase/hydrolase, acidic endochitinase pcht28-like (PR3) and monodehydroascorbate reductase 5 (MDHAR5).

**Table 1:** List of differentially expressed proteins in the resistant and sensitive pepper genotypes infested by *B. tabaci*

Biological function	Uniprot ID	Protein name	R_B:R_C	S_B:S_C	R_C:S_C
Redox regulation					
	A0A1U8ESV4	Catalase (CAT2)	**8.69**	1.84	**0.23**
	A0A1U8FQG1	Peroxidase (POD)	**6.36**	1.60	0.43
	A0A1U8EZN6	Heme-binding protein 2-like (HBP1)	3.02	0.57	**5.41**
	A0A1U8FBV9	Putative quinone-oxidoreductase homolog (CEQORH)	**2.87**	1.56	**0.39**
	A0A1U8EZE6	Heme-binding protein 2-like (HBP1)	2.69	0.89	**18.52**
	A0A1U8FMA2	Catalase (CAT)	1.98	1.27	**2.40**
	A0A1U8GY32	Glutathione S-transferase (GST)	**1.87**	1.36	1.31
	A0A1U8GBS1	Glutathione reductase (GR)	1.52	0.73	**2.89**
	A0A089FZ95	Dehydroascorbate reductase (DHAR)	1.30	0.79	**2.79**
	A0A1U8HAQ7	Uncharacterized oxidoreductase	1.29	0.78	**2.01**
	A0A1U8EL92	2-methylene-furan-3-one reductase (AOR)	1.22	0.69	**1.96**
	A0A1U8E8C1	Peroxiredoxin-2E-2(PRXIIE)	1.07	**2.40**	1.04
	A0A1U8F1N7	Peroxisomal (S)-2-hydroxy-acid oxidase (GLO1)	**0.99**	1.10	**2.00**
	A0A1U8E6R6	Monodehydroascorbate reductase (MDHAR)	0.85	**1.86**	1.08
	A0A1U8GEC0	Monodehydroascorbate reductase 5 (MDHAR5)	**0.41**	**1.91**	1.40
	A0A1U8H5G9	Superoxide dismutase (SOD)	**0.27**	1.59	**4.27**
Response to stress					
	A0A1U8FME6	Acidic endochitinase pcht28-like (PR3)	**32.37**	**14.23**	0.02
	B2CZJ6	PR10	**19.57**	1.74	**0.08**
	A0A1U8FJE1	Probable carboxylesterase (CXE6)	**10.83**	4.61	2.70
	A0A1U8E530	Annexin D4-like (ANN4)	**7.63**	1.61	0.64
	A0A1U8H0C7	Calreticulin-3 (CRT3)	**3.72**	1.37	0.73
	A0A1U8HDQ1	Flower-specific defensin-like	**3.64**	0.71	0.92
	A0A1U8FVW2	Heat shock 70 kDa protein (HSP70.1)	**2.74**	1.28	0.83
	A0A1U8EMR4	Glutamate--glyoxylate aminotransferase 2 (AOAT2)	**2.50**	1.17	1.12
	A0A1U8ELM1	Heat shock protein 90.5 (HSP90)	1.38	0.77	**1.90**
	A0A1U8GCN1	Pathogenesis-related protein STH-2-like (NUP98B)	1.38	4.33	**0.19**
	A0A1U8FJF5	CSC1-like protein ERD4(Early-responsive to dehydration stress protein (ERD4)	1.20	0.76	**1.86**
	A0A1U8EXS4	Putative amidase C869.01 (AmidP)	1.13	1.50	**0.28**
	A0A1U8E2L2	Cysteine-rich repeat secretory protein 38-like (CRRSP38)	1.05	1.26	**0.32**
	A0A1U8E6Q9	Stromal 70 kDa heat shock-related protein (CPHsp70.2)	0.97	0.87	**2.12**
	A0A1U8GL40	Stress protein DDR48-like	0.89	0.57	**0.24**
	A0A1U8EX11	Patatin	0.87	1.25	**12.23**
	A0A1U8GH17	Kirola-like	0.64	**0.17**	0.64
	A0A1U8G6G8	Chitin-binding lectin 1-like	0.54	1.12	**0.35**
	A0A1U8EN72	plasma membrane-associated cation-binding protein 1	**0.51**	0.71	1.18
	E9JEC2	Mannose-binding lectin OS=*Capsicum annuum*	0.46	1.76	**0.21**
	Q42493	Fibrillin	**0.45**	2.09	**2.89**
	A0A1U8EKU6	Myristoylated alanine-rich C-kinase substrate-like	**0.41**	1.42	1.55
	A0A1U8EZY1	Stromal 70 kDa heat shock-related protein	**0.31**	1.21	1.21
Protein metabalism and Regulation					
	A0A1U8GAJ4	Endoplasmin homolog	**2.69**	1.51	0.89
	A0A1U8G5J6	Chaperone protein ClpB1-like (HSP101)	**1.72**	0.76	0.78
	A0A1U8GVQ2	Protein disulfide-isomerase (PDIL6)	1.56	0.99	**2.53**
	A0A1U8E845	Diaminopimelate epimerase (DAPE)	1.46	1.36	**3.23**
	A0A1U8FD80	Aspartyl protease AED3	1.38	**0.19**	1.12
	A0A1U8GG27	Probable serine protease EDA2	1.33	**1.80**	1.02
	A0A1U8E900	20 kDa chaperonin, chloroplastic-like (CPN20)	1.28	0.88	**3.18**
	A0A1U8GX36	Protein disulfide-isomerase (PDIL1)	1.25	**2.21**	1.79
	A0A1U8DX55	Presequence protease 1 (PreP2)	0.98	**2.00**	1.65
	A0A1U8FIT0	Uncharacterized protein	0.85	1.41	**0.41**
	A0A1U8E5E9	60S ribosomal protein L4	**0.56**	**0.61**	1.50
	A0A1U8GMG9	60S ribosomal protein L13	**0.53**	0.58	1.40
	J7HAU1	50S ribosomal protein L2	**0.51**	0.62	1.44
	A0A1U8DRN2	50S ribosomal protein L3	**0.50**	0.76	1.29
	A0A1U8HJ31	30S ribosomal protein S20	**0.48**	0.72	**2.48**
	A0A1U8GWX0	Uncharacterized protein	**0.47**	2.88	2.59
	A0A1U8HNK6	60S ribosomal protein L7a-1	**0.43**	1.14	**2.58**
	A0A1U8ELW8	50S ribosomal protein L15	**0.40**	0.66	**2.19**
Lipid related metabolism					
	A0A1U8E9J9	Peroxisomal fatty acid beta-oxidation multifunctional protein AIM1-like	**3.54**	0.99	0.26
	A0A1U8EK22	Probable plastid-lipid-associated protein 13	1.27	0.71	**0.49**
	F2YL87	Lipoxygenase (LOX2)	1.16	**2.32**	**2.29**
	A0A1U8FRJ4	Uncharacterized protein (PLDRP1)	1.08	0.88	**2.56**
	A0A1U8F9H1	Phospholipase D (PLD)	1.00	0.88	**2.77**
	A0A1U8EAJ0	Patellin-3-like	**0.26**	1.01	1.08
Phototsynthesis related proteins					
	A0A1U8E7W8	Malic enzyme (ME1)	**4.37**	1.41	**0.36**
	A0A1U8FJN4	Oxygen-evolving enhancer protein 1	2.36	1.06	**4.79**
	A0A1U8GUM8	ATP synthase subunit b	2.19	0.75	**3.88**
	A0A1U8ESR9	Serine--glyoxylate aminotransferase (AGT1)	**2.09**	1.01	1.01
	A0A1U8FZN5	Oxygen-evolving enhancer protein 1	1.96	1.19	**2.34**
	A0A1U8FYP5	Carbonic anhydrase (CAT)	1.90	2.10	**2.26**
	A0A1U8FGM0	Photosystem II repair protein PSB27-H1	1.89	0.63	**2.45**
	A0A1U8E7H4	Ribulose bisphosphate carboxylase/oxygenase activase 1	1.86	1.16	**3.22**
	A0A1U8HDS6	Phosphoglycerate kinase (PGK2)	1.81	1.36	**1.92**
	A0A1U8GDS4	Fructose-1,6-bisphosphatase	1.75	1.07	**3.03**
	A0A1U8FUM0	ATP synthase gamma chain	1.71	0.99	**2.90**
	A0A1U8FMQ7	Ferredoxin--NADP reductase	1.61	0.74	**2.99**
	A0A1U8FRH4	Cytochrome f-like	1.43	0.97	**2.83**
	A0A1U8FHQ4	Fructose-bisphosphate aldolase	1.41	1.07	**2.31**
	A0A1U8FNB3	Transketolase (TKL1)	1.39	0.95	**2.41**
	A0A1U8E6P3	Enolase	1.32	0.85	**1.77**
	A0A1U8GVK4	Photosystem I reaction center subunit II	1.24	0.95	**2.00**
	A0A1U8HK56	LOW QUALITY PROTEIN: phosphoenolpyruvate carboxylase-like (PPC2)	1.23	**2.62**	2.31
	A0A1U8HFF2	RuBisCO large subunit-binding protein subunit beta	1.16	0.74	**2.65**
	A0A1U8FQ68	LOW QUALITY PROTEIN: photosystem II stability/assembly factor	1.14	0.91	**1.84**
	A0A1U8GZ15	Sedoheptulose-1,7-bisphosphatase	1.09	0.90	**2.20**
	K4FWQ6	Citrate synthase (CS)	1.06	1.77	**0.42**
	J7H3N5	Photosystem II protein D1	1.04	**0.51**	0.59
	A0A1U8EAE0	Oxygen-evolving enhancer protein 2	0.97	0.76	**2.48**
	A0A1U8HDT5	Porphobilinogen deaminase	0.94	0.79	**2.25**
	A0A1U8H8P7	NADP-dependent glyceraldehyde-3-phosphate dehydrogenase	0.94	1.94	**0.30**
	A0A1U8GAG1	ATP-dependent zinc metalloprotease FTSH	0.86	0.77	**1.66**
	O78327	Transketolase 1 (TKL1)	0.84	**3.24**	**2.69**
	A0A1U8EJC2	RuBisCO large subunit-binding protein subunit alpha	0.84	0.81	**2.80**
	A0A1U8EC90	magnesium-protoporphyrin IX monomethyl ester [oxidative] cyclase	**0.64**	0.68	1.39
Carbon metabolism related proteins					
	A0A1U8HHZ9	Benzyl alcohol O-benzoyltransferase	**5.54**	0.72	0.54
	A0A1U8GAR2	Cinnamoyl-CoA reductase	2.46	1.08	**2.54**
	A0A1U8FQ55	Serine hydroxymethyltransferase (SHMT)	1.22	0.99	**1.80**
	A0A1U8E7Q2	Soluble inorganic pyrophosphatase 6 (PPA6)	1.01	0.87	**3.05**
	A0A1U8F9K2	Probable Xaa-Pro aminopeptidase P	0.90	1.12	**1.89**
	A0A1U8HKG3	Glucose-1-phosphate adenylyltransferase (APL1)	0.84	0.73	**2.31**
	A0A1U8EIA9	Alpha-L-arabinofuranosidase	0.83	1.05	**0.44**
	A0A1U8F8D2	Pectinesterase	0.82	1.32	**0.22**
	A0A1U8DZQ7	Glucose-1-phosphate adenylyltransferase (APL1)	0.81	0.86	**2.06**
	A0A1U8DSA1	Uncharacterized protein	0.61	**0.22**	**0.37**
	A0A1U8F6S1	5-methyltetrahydropteroyltriglutamate-homocysteine methyltransferase	**0.51**	0.82	0.74
	A0A1U8FQ91	Xyloglucan endotransglucosylase/hydrolase (XTHs)	**0.37**	**0.22**	0.62
Other aspects					
	A0A1U8E2F0	DEAD-box ATP-dependent RNA helicase 3	**0.56**	0.83	1.07
	A0A1U8EZ49	DNA-damage-repair/toleration protein (DRT100)	1.01	**0.29**	0.46
	A0A1U8H3L5	Lysine--tRNA ligase	1.16	1.29	**0.40**
	A0A1U8H5Z0	Protein plastid transcriptionally active 16	1.15	0.90	**2.27**
	A0A1U8H847	Extracellular ribonuclease LE-like (RNS3)	2.53	1.00	**0.06**
	A0A1U8H8A4	Ribonuclease T2 family protein	0.97	2.26	**0.03**
	A0A1U8HET6	Ribonuclease S-4-like	0.96	0.94	**0.21**
	A0A1U8EW99	Protein SIEVE ELEMENT OCCLUSION:protein SIEVE ELEMENT OCCLUSION B-like (SEOR1)	0.97	1.68	**0.43**
	A0A1U8DW72	Protein EXORDIUM-like 2 (EXL2)	2.10	0.82	**3.35**
	A0A1U8HCV1	Elongation factor Tu (EF-Tu)	0.95	0.77	**2.36**

Bold indicates proteins considered as being differentially expressed at level of *p* value of ≤ 0.05

### Classification of DAPs

**Fig. 5:**
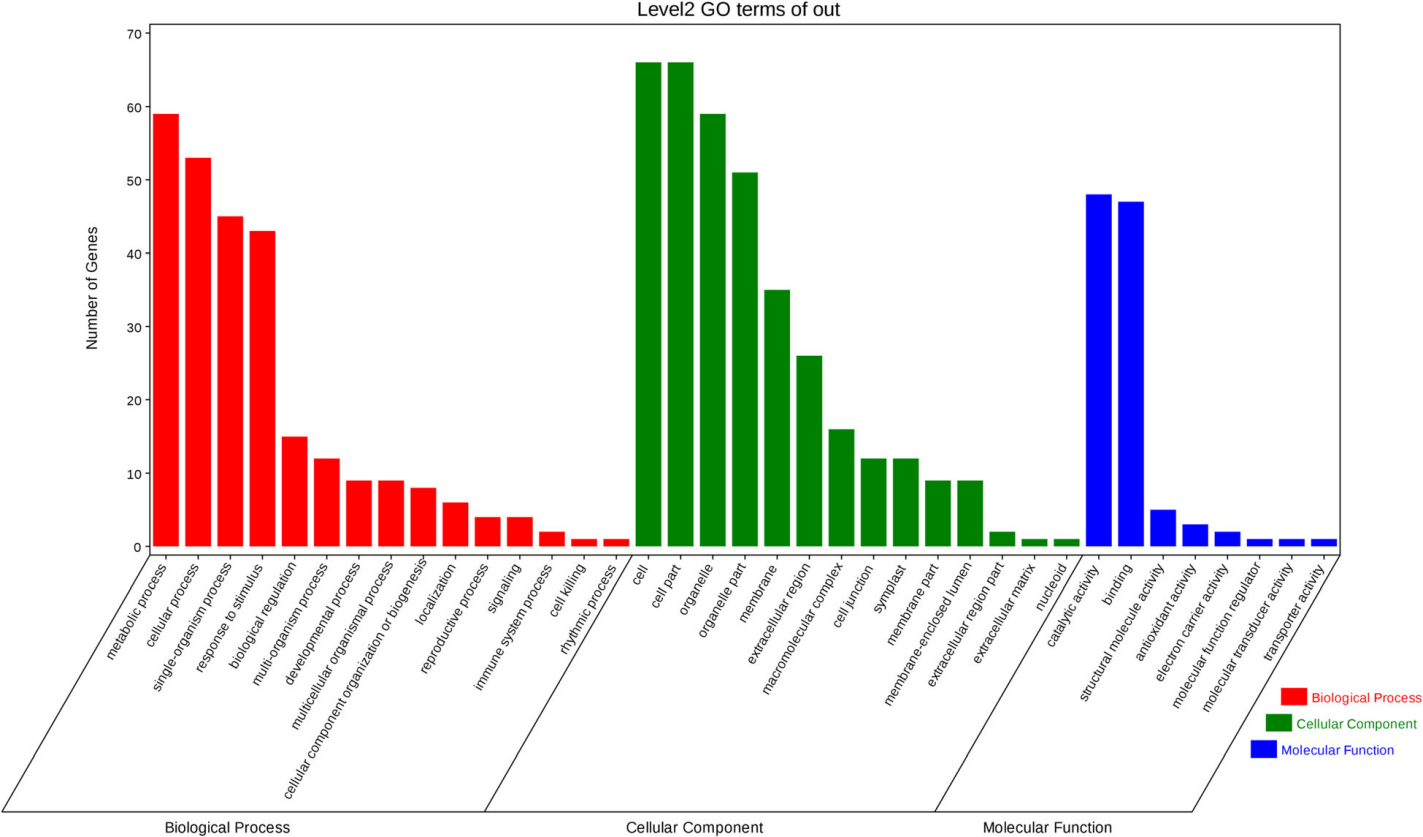
Gene ontology (GO) analysis of all differential abundance proteins (DAPs)

On the basis of gene ontology (GO) annotations, the 115 differential abundance proteins (DAPs) were grouped into three major enrichment categories using Blast2GO (Fishers exact test, FDR < 0.05). The GO results showed that 101 DAPs (90.99%) had been annotated into 37 functional groups, including 15 biological processes, 14 cellular components and 8 molecular function (Fig. 5). In the biological process, the DAPs were mainly involved in metabolic process, cellular process, response to stimulus, and so on. The ‘response to stimulus (GO:0051716)’, mainly including ‘response to oxidative stress (GO:0006979)’, existed in all of “RB-RC”, “SB-SC” and “RC-SC”, corresponding to the process of ‘hydrogen peroxide metabolic process (GO:0042743)’. Besides, a higher proportion of up-regulated ‘response to stimulus’ proteins was existed in “RB-RC” (Additional file 6: Table S3). Furthermore, more photosynthesis-related protein changes existed in “RC-SC”, such as photosynthesis (GO:0015979), plastid organization (GO:0009657), and light reaction (GO:0019684) (Additional file 6: Table S3). In the molecular function category, the DAPs were mainly involved in catalytic activities and binding (Additional file 7: Table S4). In addition to these two major categories, additional categories identified corresponded to structural molecule activities (GO:0005198), antioxidant activities (GO:0016209), electron carrier activity (GO:0009055), and so on. A higher proportion of ‘hydrolase activity (GO:0004553)’ proteins was found in “RB-RC” and “SB-SC” (Additional file 7: Table S4). At the cellular component category, though ‘extracellular region’ existed in all of “RB-RC”, “SB-SC” and “RC-SC”, a higher proportion of increased proteins existed in “RB-RC” and “RC-SC”, indicating that the related extracellular proteins including cell wall proteins are changed to improve tolerance to *B. tabaci* infestation (Fig. 5; Additional file 8: Table S5). Besides, ‘chloroplast’ also existed in the three ratio parameters, but the higher proportion of increased proteins in chloroplast existed in “SB-SC” and “RC-SC” (Additional file 8: Table S5), suggesting that photosynthesis-related proteins play an important role in pepper resistance to *B. tabaci*.

For further functional categorization, KEGG pathway analyses were performed using the KOBAS3.0 database and all DAPs were assigned to 16 KEGG pathways (*P* < 0.05). All of the proteins in KEGG categories were shown in Additional file 9: Table S6. In “RB-RC”, DAPs were assigned to ribosome (ko03010), peroxisome (ko04146), protein processing in endoplasmic reticulum (ko04141), and so on, whereas DAPs were assigned to ascorbate and aldarate metabolism (ko00053) and carbon fixation (ko00710) in “SB-SC”. In “RC-SC”, it was found that the DAPs participated in the resistant related pathways, such as photosynthesis (ko00195), carbon fixation (ko00710), RNA degradation (ko03018), glycolysis/gluconeogenesis (ko00010), peroxisome (ko04146) and linoleic acid metabolism (ko00591). Pathways common to “RB-RC”, “SB-SC” and “RC-SC” include carbon fixation and linolenic acid metabolism. To gain more insight into photosynthesis-related protein for tolerance to *B. tabaci*, the DAPs of “RB-RC”, “SB-SC” and “RC-SC” were analyzed in Additional file 1: Figure S1 and Additional file 2: Figure S2. Based on the GO function and KEGG pathway analysis, multiple proteins involved in stimulus response, antioxidant defense, photosynthesis and linoleic acid metabolism may play defensive role against the *B. tabaci* damage. A master table (Table 1) that summarized all changed proteins was generated to obtain an overview of the proteins in response to *B. tabaci*.

### Validation of iTRAQ data for selected proteins by PRM

**Fig. 6:**
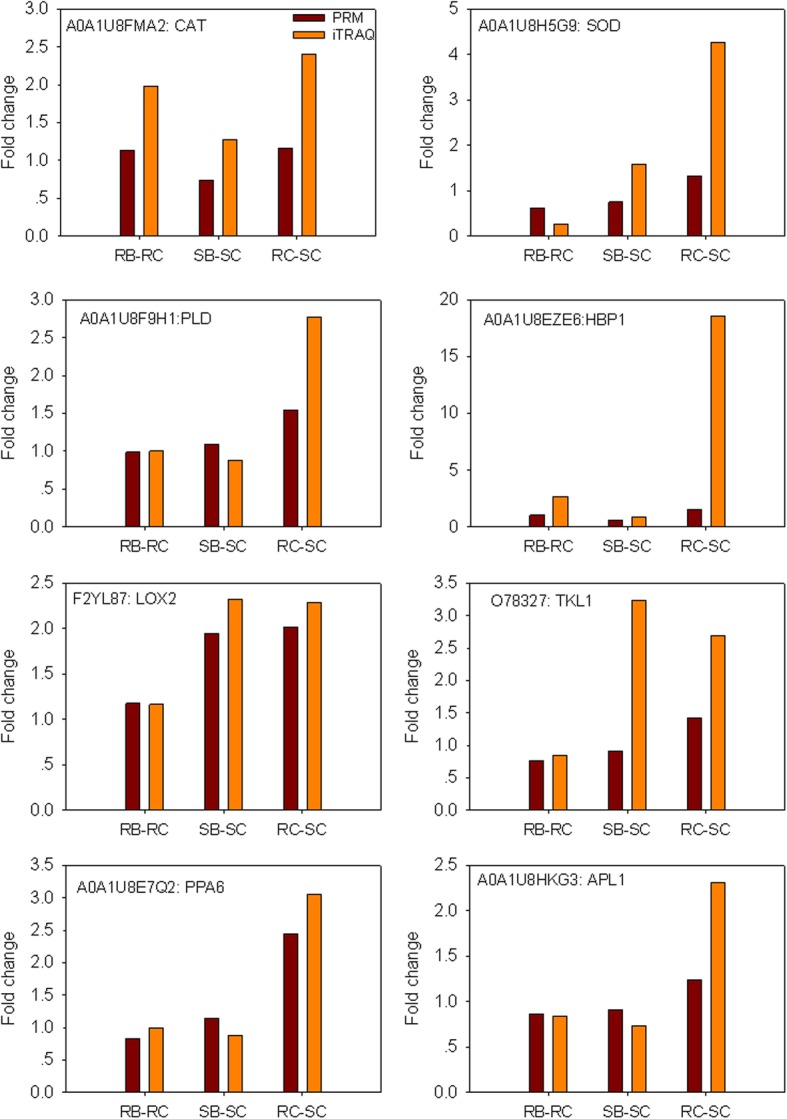
Relative expression levels of selected proteins measured by PRM in the RB-RC, SB-SC and RC-SC. RB-RC represents protein level changes in the resistant genotype after *B. tabaci* infestation; SB-SC represents protein level changes in the susceptible genotype after *B. tabaci* infestation; RC-SC represents protein level changes in the resistant/susceptible genotype under control conditions. The protein samples for PRM were exacted from peppers treated with *B. tabaci* for 48 h

PRM is a recent development in targeted mass spectrometry, which is more specific and sensitive than selected reaction monitoring and has been widely used to quantify and detect target proteins [15, 16]. In this study, the protein expression levels obtained by iTRAQ were confirmed by quantifying the expression levels of some proteins by PRM-MS analysis. Ten candidate proteins related to resistance to *B. tabaci* were selected for PRM analysis. Among 10 target proteins, 8 proteins have MS/MS spectrum(s) and unique peptide(s). Therefore, the PRM detection was performed for the 8 protein only (CAT, SOD, PLD, HBP1, LOX2, TKL1, PPA6, APL1). In general, the trends in the change of the results measured by PRM and iTRAQ are basically consistent (Fig. 6). However, there is difference between the actual value. The difference between the values may be due to the different detection methods [16, 17]. Therefore, our iTRAQ results are reliable and reproducible.

### Confirmation of iTRAQ data for selected proteins by qRT-PCR

**Fig. 7:**
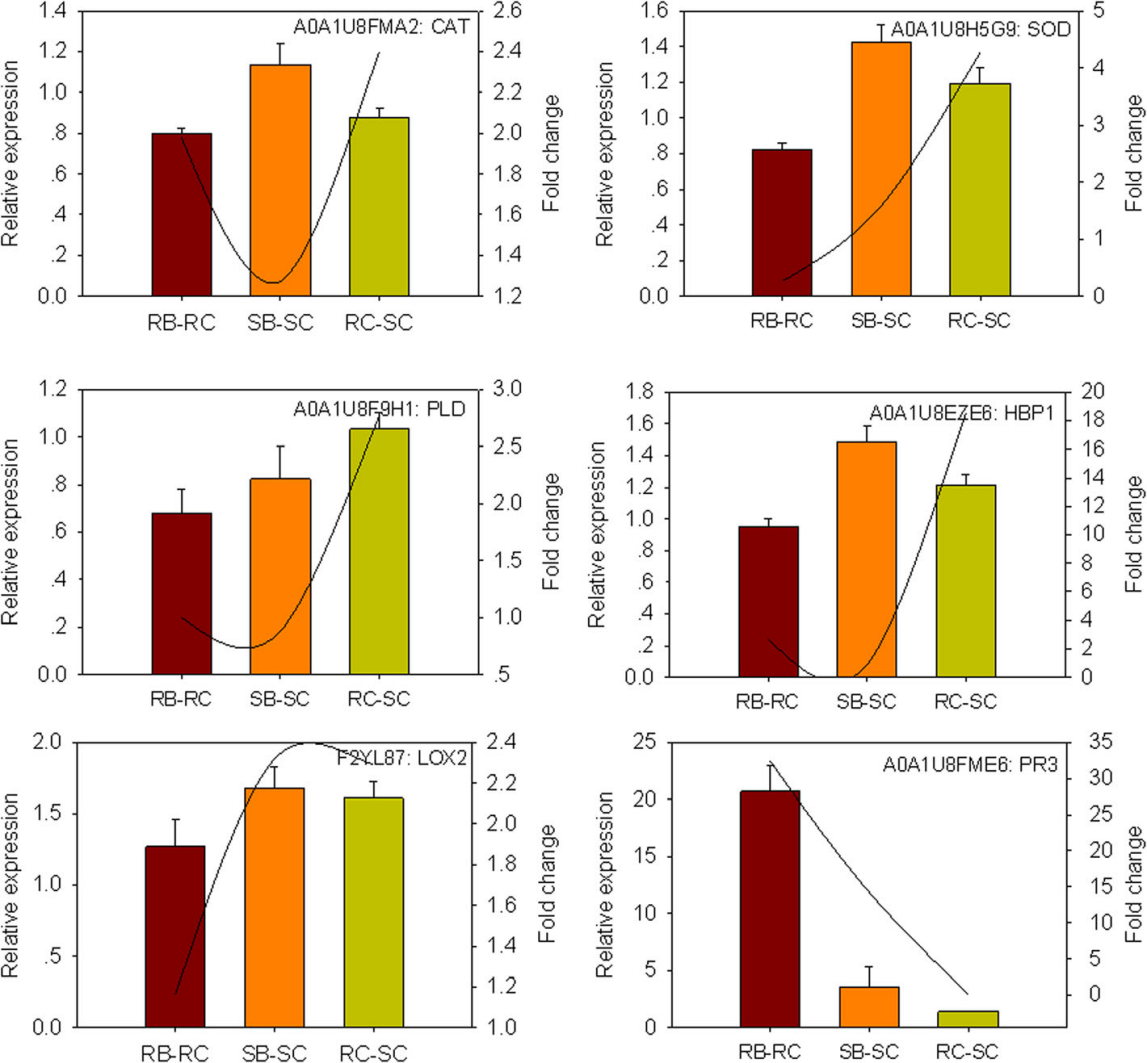
Real-time PCR analysis of genes encoding the selected proteins in RB-RC, SB-SC and RC-SC. RB-RC represents protein level changes in the resistant genotype after *B. tabaci* infestation; SB-SC represents protein level changes in the susceptible genotype after *B. tabaci* infestation; RC-SC represents protein level changes in the resistant/susceptible genotype under control conditions. The expression levels of *CAT*, *SOD*, *HBP1*, *LOX2*, *PLD* and *PR3* were quantified relative to the value obtained from control samples (*B. tabaci*-free plants). The column means the relative expression level of genes and the line means the trend value of iTRAQ

To further confirm the iTRAQ data, we monitored the expression patterns of the corresponding genes encoding proteins using qRT-PCR. The expression patterns of the six genes (*CAT*, *SOD*, *PLD*, *HBP1*, *LOX2*, *PR3*) are shown in Fig. 7. The expression trends of four genes basically matched with our iTRAQ data, except for the other two genes (*CAT* and *HBP1*). The low correlation coefficient of the proteome and transcriptome data has been reported previously [18, 19]. The discrepancies could potentially be attributed to mRNA stability, splicing, translational regulation, post-translational processing, control of protein turnover, protein degradation or a combination of these [20].

## Discussion

### Generation of a comprehensive proteome map of *B. tabaci* infested pepper

*B. tabaci* is a major pest of both greenhouse and open-field horticultural crops. Screen and utilization of resistant plants to control *B. tabaci* is an important mean of agricultural production. To date, our understanding of the molecular mechanism underlying the defense response of plants to *B. tabaci* is limited, particularly of resistant plants. Proteomics has emerged as a powerful tool to explore physiological changes at the cellular level, but few attempts have been made to study the response of pepper to *B. tabaci* attack at the level of proteome. Yin et al. [6] showed the proteome change of *A. thaliana* leaves infested by *B. tabaci* using two-dimensional electrophoresis. In that report, however, a single Arabidopsis cultivar was used for *B. tabaci* infestation and only 20 proteins were generated. In the present study, the distinct genetic background of RG and SG genotypes provided a solid foundation for identifying proteins involved in the pepper defense response against *B. tabaci* attack. To our knowledge, it is the first time that the advanced proteomic technology (such as iTRAQ) was used to study *B. tabaci* resistant mechanisms using two different resistant materials.

Two methods were utilized to fully assess the iTRAQ differential expression data (Additional file 10: Table S7). For the first one, a 1.50-fold or 0.67-fold change threshold with a *P*-value < 0.05 in the average value of three replicates was classified as a physiologically significant change. A total 398 DAPs were identified in the three classes of differentially accumulated proteins of “RB-RC” (130), “SB-SC” (139) and “RC-SC” (251). To further search for key protein components or pathways for *B. tabaci* resistance response, a 1.50-fold or 0.67-fold change threshold with a *P*-value < 0.05 in at least two experiments and in the average value of three replicates were classified as a significant change. A total 115 DAPs were identified in the three classes of differentially accumulated proteins of “RB-RC” (37), “SB-SC” (17) and “RC-SC” (77). More DAPs were present in the RB than SB. The results are supported by previous studies reporting that *B. tabaci* infestations drive more differentially expressed genes (DEGs) in the strong resistance cultivar than sensitivity [13] Besides, differential expression analyses revealed that more up-regulated proteins were identified than down-regulated in the classes of “SB-SC” and “RC-SC”. However, our results contrast with that observed in cotton stressed by *B. tabaci*, in which down-regulation > up-regulation of DEGs. The inconsistence in proteins and genes may explain the discrepancy in the translational and post-translational regulations in pepper defense against *B. tabaci* infection. In the present study, the function of DAP from the second method was further analyzed and discussed in the following sections. GO enrichment and KEGG pathway analysis indicated that proteins involved in processes such as oxidative stress regulation, stimulus response, linoleic acid metabolism and photosynthesis might be involved in the host plant resistance to *B. tabaci* infestation.

### DAPs involved in oxidative stress

Reactive oxygen species (ROS) are generated in plant tissues in response to different stresses including the interaction with herbivores [21]. ROS are important signal molecules in plants, but can also cause plant toxicity. Plants have formed a set of enzymatic systems to scavenge highly ROS. In this study, several oxidative stress-related proteins such as catalase (CAT, A0A1U8FMA2), peroxidase (POD, A0A1U8FQG1), superoxide dismutase (SOD, A0A1U8H5G9), dehydroascorbate reductase (DHAR, A0A089FZ95), glutathione reductase (GR, A0A1U8GBS1) and monodehydroascorbate reductase 5 (MDHAR5, A0A1U8GEC0) were identified in response to *B. tabaci* attack (Table 1).

SOD is the first enzyme with free radicals as the substrate, catalyzing the dismutation of superoxide radicals to O_2_ and H_2_O_2_, maintaining adequately low oxyradical levels. It is an important protective enzyme in plants’ cell defense systems, and is closely related to the plants’ resistances [17]. In this study, the SOD was up-regulated in RC when compared to SC, indicating that the higher resistance materials may display high ability to scavenge ROS. CAT catalyzes decomposed H_2_O_2_ to water and oxygen and CAT are characterized by the oxidation of various organic compounds, which have been shown to be involved in insect response in rice [20]. PODs are one kind of essential enzymes of the immediate response of plants to insect damage. The role of PODs in plant resistance to insect pests has been studied in various plant systems. For example, production of phenoxy and other oxidative radicals by PODs in association with phenols directly deters the feeding by insects and/or produces toxins that reduce the plant digestibility, which in turn leads to nutrient deficiency in insects with drastic effects on their growth [22]. In this study, the iTRAQ data showed that the expression levels of CAT and POD were induced in both pepper genotypes, but were only significantly induced in the RB. It is speculated that both proteins in the resistant pepper have the ability to reduce the ROS damage caused by *B. tabaci*. MDHAR, DAR and GR are the key enzyme in the ascorbate acid- glutathione (AsA-GSH) cycle [23]. AsA-GSH cycle is considered to be an important mechanism for resistance of plants under stress conditions [24]. In this cycle, ascorbate peroxidase (APX) catalyzes the reduction of H_2_O_2_ into water with AsA serving as an electronic donor. DHAR utilizes the electrons provided by GSH to reduce DHA, while DHA is previously produced from MDHA. Simultaneously, GSH is oxidized into glutathione disulfide (GSSG) by DHAR and GSSG is then reduced into GSH, catalyzed by GR. Greenbug feeding on resistant sorghum induced the expression of peroxidase and glutathione-S-transferase genes, but both up- and down-regulated different CAT genes [25].

Arabidopsis MDARP and MDAR3 were also up-regulated by *B. tabaci*, which may function to scavenge excessive ROS that result from *B. tabaci* feeding [6]. Here, three differentially expressed enzymes MDAHR, DAR and GR were detected, which were all up-regulated in RC when compared to SC, indicating that the high level of AsA-GSH cycle plays an important role in the protection of pepper seedlings against *B. tabaci* injury. Thompson and Goggin [26] showed that phloem-feeding insects (PFIs) did not uniformly regulate the oxidative stress-related genes in whole leaf tissues. Our results also showed that the *B. tabaci* triggered a differential modulation of antioxidant proteins in both genotypes. For example, *B. tabaci* infestation up-regulated the expression of CAT and POD, but down-regulated the expression of SOD and MDAHR in the resistant pepper.

Besides, under oxidative stress conditions the lipid constituents of cells can undergo oxidation, which is toxic to biomolecules and several enzyme activities. Recently, CeQORH was reported to reduce the double bond of stress-related oxidized lipids named γ-ketols [27]. In the present study, A0A1U8FBV9 (CeQORH) is a chloroplast envelope quinone oxidoreductase homolog, which was induced by *B. tabaci* in the SC (Table 1). Haem is prominent among the iron binding molecules in the cell. The presence of free haem in the cytoplasm must be maintained at a low concentration to prevent oxidative stress through the oxidation of haem iron [28]. A0A1U8EZE6 and A0A1U8EZN6 (HBP) are heme-binding protein-like protein, which were induced by *B. tabaci* in the RB, but not in SB, and had a higher level in the RC compared to the SC. Early researches have confirmed that Arabidopsis homologous gene *AtHBP5* was involved in antioxidant pathway. *AtHBP5* over-expressing plants show a decreased accumulation of H_2_O_2_. It is proposed that the interaction between the HY1 and AtHBP5 proteins participate in an antioxidant pathway that might be mediated by reaction products of haem catabolism [29]. Oxidative stress is one of the first general reactions to the injuries caused by insects when they penetrate the plant [20]. Our results suggest that the resistant pepper has a high ability to cope with the oxidative damage and *B. tabaci*-triggered differential regulation of antioxidant proteins is possibly due to different degrees of disruption of cell redox homeostasis.

### DAPs involved in stress response

Plants are endowed with constitutive and inducible protective mechanisms of biotic−/abiotic-defense known as the stress or defense responses [30, 31]. In the present study, several stress-related proteins were differentially regulated in both genotypes, among which, three proteins are HSP type (A0A1U8E6Q9, A0A1U8ELM1, A0A1U8FVW2). A0A1U8E6Q9 is a stromal 70 kDa heat shock-related protein and its homologous protein, Arabidopsis CPHsp70.2, is required for protection against oxidative stress in *Arabidopsis thaliana* [32]. A0A1U8ELM1 is a heat shock protein 90.5, which is a chloroplast localized HSP90 family molecular chaperone in Arabidopsis, and it has been implicated in plant abiotic stress resistance, photomorphogenesis and nuclear-encoded protein import into the chloroplast [33]. A0A1U8FVW2 is a heat shock 70 kDa protein (Hsp70–1), and cytosolic Hsp70s have been shown to be involved in the thermotolerance and the immune response in Arabidopsis, and be required for productive potyvirus infection of tobacco plants [34]. From our iTRAQ data, CPHsp70.2 and Hsp90.5 showed significant accumulation (about 2-fold) in RC-SC, but Hsp70–1 was only upregulated in the RB. The change in the expression profile indicates that these HSPs are involved in *B. tabaci* tolerance.

Pathogenesis-related proteins (PR proteins) and defense-related proteins are specifically induced under stress conditions [31]. Three biotic stress-related proteins were identified as PR protein (A0A1U8FME6, A0A1U8HDQ1, B2CZJ6), of which the expression level in the RB were higher than that in the SB (Table 1). A0A1U8FME6 is one kind of PR3 protein, encoding a basic chitinase involved in ethylene/jasmonic acid mediated signaling pathway during systemic acquired resistance. PR3 genes were induced systemically but not locally and they can be candidates for broad-spectrum resistance, viz., induced systemic resistance [35]. A0A1U8HDQ1 encodes a member of a family of small, secreted, cysteine rich protein with sequence similarity to the PCP (pollen coat protein) gene family. B2CZJ6 encodes a member of the PYR (pyrabactin resistance)/RCAR5 (regulatory components of ABA receptor) family proteins, which is also known as PR10 [36]. Overexpression of RCAR5 resulted in ABA-hypersensitive phenotypes and enhanced the resistance of Arabidopsis plants to *Pseudomonas syringae* pv. tomato (Pst) DC3000, through promoting stomata closure leading to the development of resistance to this bacterial pathogen [37]. PR proteins not only inhibit pathogen progress but also help in host plants growth, which accumulate locally in the infected and surrounding tissues. Production of PR proteins in the uninfected plant’s parts can prevent the affected plants from further infection [35]. These results suggest that these PRs may be involved in plant perception and responses to *B. tabaci* attack signals.

Other stress-related proteins like annexin D4-like (ANN4, A0A1U8E530) and calreticulin-3 (CRT3, A0A1U8H0C7) were increased in the leaves when the *B. tabaci* were feeding on the pepper, of which the expression was significantly increased in the RB than that in the SB. ANN4 encodes Ca^2+^-regulated membrane-binding proteins modulating cytosolic calcium signatures. Huh et al. [38] provided experimental evidence that *AnnAt4* and *AnnAt1* interact with each other in a Ca^2+^-dependent manner and function to regulate responses to drought and salt stress. Recently, ANN4-mediated cytosolic calcium signaling was reported to be involved in MYB30-regulating oxidative and heat stress responses in Arabidopsis [39]. In our experiment, ANN4 expression was significantly induced in the RB, reaching 7.63-fold, but remained unchanged in the SB, indicating that ANN4 and ANN4-mediated calcium signaling may be involved in pepper resistant to *B. tabaci* damage. CRT has been documented to be a Ca^2+^-binding molecular chaperone that facilitates the folding of newly synthesized glycoproteins and regulates the Ca^2+^ homeostasis in the endoplasmic reticulum (ER) lumen [40]. It has been suggested that Arabidopsis CRT3 mediate plant defense against viral and biotrophic pathogens [41] Arabidopsis *CRT* mutant *atcrt3* is more sensitive *Pseudomonas syringae* pv tomato DC3000 (Pst DC3000) and water stress [40, 41]. Therefore, a high expression of CRT3 in the highly resistant pepper may be determined to be a defense against *B. tabaci* invasion.

### DAPs involved in protein metabolism and regulation

When plants are attacked by insects, they produce many defense-related proteins, many of which are synthesized and then secreted to their various destinations within the cell [20]. In terms of protein synthesis, six ribosomal proteins such as 30S ribosomal protein S20, 50S ribosomal protein L2/L15 and 60S ribosomal protein L4/L13/7a-1, showed higher abundance in the RC than in the SC. However, these ribosomal proteins were down-regulated in the RB, but remained unchanged in the SB. The results indicate that protein synthesis processes in resistant plants can maintain a high level, although the exact influencing mechanism is still unclear.

Protein disulfide isomerase (PDI) is a member of the thioredoxin superfamily, and is involved in the progression and maturation of secretory proteins in the ER [42, 43]. AtPDI6 acts as an attenuator of D1 synthesis, modulating photo inhibition in a light-regulated manner. PDI is a component of unfolded protein response that alleviates ER stress and lessens programmed cell death [44]. Recently, it is suggested that PDIs serve both specialized and overlapping functions to adapt to new biochemical needs or environments. Peng et al. [43] showed that transgenic tobacco overexpressing *AtPDI6* was more tolerant to high concentrations of 2,4,6-Trichlorophenol (TCP) implying that AtPDI6 can be used for TCP detoxification by the way of overexpression in plants. In our experiments, two PDIs (PDIL6 A0A1U8GVQ2) and (PDIL1 A0A1U8GX36) showed higher abundance in the RC than in the SC without *B. tabaci* invasion. The level of PDIL1 increased more than 2-fold in the SB-SC but not in the RB-RC after *B. tabaci* invasion. All results suggest that the resistant peppers have strong basal defense through regulating ribosomal proteins and protein disulfide isomerases.

A well-defined response of plants to stress involves the enhanced production of heat shock proteins, which maintain the cellular proteostasis in limiting the production and accumulation of protein aggregates induced by stress, thereby contributing to restoring cellular protein homeostasis disrupted by stress conditions [45–47]. In the previous section, the functions of CPHsp70.2, HSP90 and Hsp70–1 has been discussed. Recently, it appears that some of these HSPs are capable of controlling the mRNA translation under normal or stress conditions [47, 48]. Small HSPs and HSP101 are involved in the resolubilization of translation factors like eEF1B and eIF4A during the recovery phase [46, 47]. Merret et al. [47] showed that HSP101 is required for the efficient release of ribosomal protein mRNAs from stress granules resulting in a rapid restoration of the translation machinery by producing new ribosomal proteins. In our study, A0A1U8G5J6, a chaperone protein ClpB1-like protein, homologous to Arabidopsis HSP101, increased 1.72-folds in response to *B. tabaci* attack in the RB but maintained no change in the SB (Table 1). These observations suggest that the resistant genotype can rapidly adjust translational levels following *B. tabaci* stress.

### Lipid metabolism-related proteins

Lipid-mediated signal processes are crucial for cell survival, growth and differentiation and for plant responses to biotic and abiotic cues such as salinity, pests, and pathogens [49]. Signaling lipids include a wide range of lipid classes, such as lysophospholipid, fatty acid and phosphatidic acid. In the present study, AIMI (A0A1U8E9J9), a peroxisomal fatty acid beta-oxidation multifunctional protein, was increased in the RB. AIMI is essential for seedling establishment and is also involved in JA biosynthesis [50]. Phospholipase D (PLD) hydrolyzes common membrane phospholipids, to generate a free head group and phosphatidic acid (PA). The PLD-mediated hydrolysis of phospholipids is highly prominent in plants and play important roles in plant response to stress, including plant-pathogen interactions [51]. In our experiment, PLD alpha 1 (A0A1U8F9H1) and PLDrp1 (A0A1U8FRJ4) were not induced by *B. tabaci* attack in both genotypes, but the expression levels of them were higher in the RC than that in the SC (about 3.0-fold). Arabidopsis mutants with antisense suppression of *PLDα* expression decreased PA and JA production. PLD has been implicated in JA/oxylipin formation in plant interaction with *Botrytis cinere* and virulent Pst DC3000 [52]. The function of PLDrp1 remains unknown, but its abundant expression and distribution in plants suggest that it binds PA and acts downstream of PLDα pathway. The PA-binding phosphoprotein PLDrp1 is regulated by PLDα1 in a stress-dependent manner [53]. Our results suggest that PLD-mediated signals in SB help improve resistance against *B. tabaci* attack, though the expression of PLDrp1 and PLDα1 were not activated by *B. tabaci*.

JAs play a central and conserved role in promoting resistance to a broad spectrum of insects, which are lipid-derived signals originating from α-linolenic acid in chloroplast membranes [50]. Plant lipoxygenases (LOXs) catalyze the oxidation of polyunsaturated fatty acids, generating hydroperoxy fatty acids. LOX2 encodes a 13(S)-lipoxygenase (LOX), that control the first dedicated step in the biosynthesis of JAs, catalysing the initial step of α-linolenic acid into (13S)-hydroperoxyoct adecatrienoic acid. In this study, the LOX2 (F2YL87), homologous to Arabidopsis AtLOX2 was increased significantly in the SB, but not in the RB. In rice, overexpressing *OsLOX* plants increased endogenous levels of JA, showing reduced plant mortality when infested with the phloem-feeding brown plant hopper [54]. In maize, ZmLOX10, a 13-LOX was involved in resistance against chewing *Spodoptera exigua* larvae [55]. In barley, *LOX2.2* overexpressing lines showed up-regulation of some other JA-regulated genes with lower aphid number, suggesting that LOX2.2 plays a role in the activation of JA-mediated responses and indicates the involvement of LOX2.2 in basic defense responses [56]. Recently, it is reported that LOX2 is involved in green leaf volatiles (GLVs) biosynthesis in Arabidopsis [57]. GLVs have been shown to induce defense responses and are involved in indirect defense in plant-insect interactions [58, 59]. It is also reported that LOX2 is involved in singlet oxygen generation as a response to wounding induced by herbivore as well as by physical factors, which provide novel insight into wound-induced signaling in the local defense reaction [60]. Though LOX2 was only induced by *B. tabaci* in the SB, RC has a higher level of LOX2 compared with SC under normal conditions. The *B. tabaci*-regulated LOX2 expression was further confirmed by PRM and RT-qPCR (Fig. 6 and Fig. 7). Thus, the susceptible pepper plants seem to activate their basal defense mechanism of LOX2-mediated JA signal under *B. tabaci* attack, which has already existed and maintained a high level in the resistant plants.

### DAPs involved in photosynthesis

In addition to the herbivore-induced production of physical and chemical defenses, numerous changes in plant primary metabolism occur in response to insect herbivores [61]. Research on changes in primary metabolism associated with insect feeding has been focused largely on the role of carbohydrates as products of photosynthesis. In the present study, KEGG enrichment analysis has revealed that eight proteins (A0A1U8GUM8, A0A1U8FUM0, A0A1U8FRH4, A0A1U8GVK4, A0A1U8FGM0, A0A1U8FJN4, A0A1U8FZN5 and A0A1U8EAE0) are involved in photosynthesis system (Additional file 1: Figure S1) and seven proteins (A0A1U8FNB3, A0A1U8HDS6, A0A1U8E7W8, A0A1U8GDS4, A0A1U8GZ15, A0A1U8HK56, A0A1U8FHQ4) are involved in carbon fixation in photosynthetic organisms (Additional file 2: Figure S2). Besides, A0A1U8FNB3, A0A1U8GZ15, O78327, A0A1U8E7H4, A0A1U8EJC2 and A0A1U8HFF2 were also involved in the photosynthetic CO_2_ fixation process. For example, Chloroplast TKL (transketolase, A0A1U8FNB3; O78327) is a key enzyme of plant carbon metabolism due to its amphibiotic role in both the Calvin–Benson–Bassham (CBB) cycle and the oxidative pentose phosphate pathway [62]. Interestingly, most of proteins’ expression level in the RC were significantly higher than those in the SC, but these proteins were not significantly increased in the RB and SB (Table 1).

There are two conflicting views on how plants should alter photosynthesis, and thereby carbon fixation, to optimize defense. Photosynthetic activity might be promoted or be reduced by herbivory [61]. For example, Bilgin et al. [63] showed that biotic stress globally down-regulates photosynthesis genes. While Halitschke et al. [64] showed herbivore-specific elicitation of photosynthesis by mirid bug salivary secretions in the wild tobacco. In this study, the idea that increased photosynthesis is highly resistant to *B. tabaci* was supported by photosynthesis-related protein expressions using iTRAQ method. Previously, wheat and barley resistance to Russian wheat aphids has been associated with increased expression of photosynthesis-related genes after *B. tabaci* attack [65, 66]. Photosynthetic activity could promote because (1) synthesis of defensive metabolites requires carbon fixation or (2) increasing photosynthetic activity to compensate for the loss of leaf area by insects [61]. The results showed that the resistant material had high photosynthesis ability and can respond to *B. tabaci* attack, suggesting that photosynthesis and carbon metabolism might be involved in the resistance of pepper to *B. tabaci*.

Some other proteins were also found involved in plant defense. For example, SEOR1 is a phloem filament protein involved in plant defense [67]. Aphids of the species *Myzus persicae* on *Arabidopsis thaliana AtSEOR1* and *AtSEOR2* mutants perform worse when compared to aphids on control plants, indicated by reduced reproduction and shortened reproduction period. However, Anstead et al. [68] concluded that SEOs were not involved in plant defense against phloem-feeding insects. Pagliari [69] showed that the low phytoplasma titer was found in *AtSEOR1* mutant lines indicating the possible involvement of this gene in plant defense mechanism. Besides, a possible role of AtSEOR1-mediated JA and cis-12-oxo-phytodienoic acid metabolisms was observed in plant defense against phytoplasmas. PGK2, a nucleus-encoded chloroplast phosphoglycerate kinase, plays a central role in cell metabolism. PGK protein is required for efficient watermelon mosaic virus (WMV, genus Pot virus) infection in the Arabidopsis [70].

## Conclusions

In the present study, an overview of the protein expression profile in the pepper resistant genotype and sensitive genotype in response to *B. tabaci* attack at 48 h was first explored by iTRAQ technique. The proteomic data presented here will help us to further understand the molecular mechanisms of plant resistance to *B. tabaci*. It is suggested plant express more redox regulation-related proteins to deal with the oxidative damage caused by *B. tabaci* to improve plant tolerance. The PR3, Hsp70–1, HSP101 and JA pathway were more active in the resistant genotype, which might contribute to *B. tabaci* resistance in pepper. Meantime, our results support the view of increased photosynthesis (carbon metabolism) is involved in the resistant of pepper to *B. tabaci*. Besides, ANN4, CRT3, CEQORH and AIMI were specifically found here involved in *B. tabaci*–pepper interaction processes. In the future, studies on the function of specific proteins found in this study will be helpful to explore the mechanisms of host resistance to *B. tabaci* attack.

## Methods

### Plant materials and insect maintenance

In this study, the pepper (*Capsicum annuum*) genotype xinsujiao No.15 (resistant genotype, RG) and the genotype sujiao No.15 (susceptible genotype, SG) were acquired from the Vegetable Institute, Jiangsu Academy of Agricultural Sciences, Nanjing, China. Seeds were grown in a pest-free growth chamber at Yangzhou University, China. The plants were irrigated and fertilized according to horticultural practices but without spraying herbicides. A virus free colony of *B. tabaci* biotype B used for infestation was maintained on tomato in greenhouse of insectary.

### Non-preference test

Two resistant pepper seedlings and two susceptible seedlings at 7th leaf stage with approximately the similar leaf area were selected and placed in a 60-mesh gauze cage to prevent the whitefly escape. Four pots of seedlings were arranged in a circle. 200 adult insects were collected in a container and hung into the center of the circle. The number of adults on each seedling was counted 24 h, 48 h and 72 h after release, and the average number of insects settled on each variety was determined based on the number of insects on the 72-h seedling. The experiments were performed in 5 biological replicates and repeated twice to confirm the results.

### Egg hatchability

For egg hatchability test, the 7th leaf stage healthy seedlings (cv. RG and SG) were selected. Each plant was covered with 60-mesh gauze and infested with five pairs of newly emerged adult *B. tabaci*. At 3 days after release, the eggs (including nymphs) in every seedling were counted. The experiments were performed in five biological replicates and repeated twice to confirm the results.

### *B. tabaci* infestation assay

The tested samples (cv. RG and SG) were grown to the 7th leaf stage. 50 adult insects were collected and released onto the 5th leaf and closed with a small ventilate pocket (Additional file 3: Figure S3). The plants grown as others without the insect infestation were set as control. After *B. tabaci* infestation for 48 h, the infected leaves were cut and frozen immediately in liquid nitrogen, and store at − 70 °C refrigerator for further use. All experiments were performed in three biological replicates.

### Protein extraction and reductive alkylation treatment

The design of proteomic study is shown in Fig. 1. For protein extraction, twelve leaf samples (three biological replicates of two genotypes for the control and *B. tabaci* infestation; Treatment: SB and RB mean SG pepper and RG pepper with *B. tabaci* infestation, respectively; Control: SC and RC refer to SG pepper and RG pepper without *B. tabaci* infestation, respectively) were individually ground to powder in liquid nitrogen, and incubated in lysis buffer (7 M Urea, 2 M Thiourea, 4% SDS, 40 mM Tris-HCl, pH 8.5) containing 1 mM PMSF and 2 mM EDTA (final concentration) for 5 min, then 10 mM DTT (final concentration) were add to the sample. The samples were sonicated for 15 min and centrifuged at 4 °C, 13,000×g for 20 min. The supernatant was transferred to a new tube and mixed with 4 volumes of precooled alkylation at − 20 °C overnight. After centrifugation, the protein pellets were air-dried and resuspended in 8 M urea/100 mM TEAB (pH 8.0). Protein samples were reduced with 10 mM DTT at 56 °C for 30 min, and alkylated with 50 mM iodoacetamide (IAM) for 30 min in the dark.

### Trypsin digestion and iTRAQ labeling

After diluted 5 times with 100 mM TEAB, 100 μg of proteins from each sample were used for tryptic digestion. Trypsin (Promega, Madison, WI, USA) was added at an enzyme-protein ratio of 1:30 (w/w), and digested at 37 °C overnight. The digested peptides were acidified using equal volume of 0.1% formic acid (FA) solution and desalted with Strata-X C18 column. The acidified enzymatic hydrolysate was injected to column for three times, then washed the column with solvent A (0.1% FA in 5% ACN) twice, the peptides were eluted 1 ml with solvent B (0.1% FA in 80% ACN). Finally, peptides were lyophilized and reconstituted in 20 ul 0.5 M TEAB for peptides labeling with iTRAQ 4-plex kits (AB Sciex Inc., USA) according manufacturer’s protocol: Control and *B. tabaci* treatment of two pepper genotypes were labeled respectively as Additional file 11: Table S8 shown. The labeled samples were combined and lyophilized. Next, labeled samples were fractionated using high-performance liquid chromatography (HPLC) system (Thermo DINOEX Ultimate 3000 BioRS) with a Durashell C18 (5 um, 100 Å, 4.6x250mm) and 12 fractions collected.

### LC-ESI-MS/MS analysis

Each fraction was dissolved in 30 μl of 2% acetonitrile and analyzed using Triple TOF 5600+ mass spectrometer coupled with the Eksigent nanoLC System (SCIEX, USA). 5 ml of peptide sample was loaded onto a C18 trap column (5 μm, 100 μm × 20 mm), and eluted at 300 nL·min^− 1^ onto a C18 analytical column (3 μm, 75 μm × 150 mm) over a 90 min gradient. The two mobile phases were buffer A (2% acetonitrile/0.1% formic acid/98% H_2_O) and buffer B (98% acetonitrile/0.1% formic acid/2% H_2_O). For IDA (information dependent acquisition), survey scans were acquired in 250 ms and 30 production scans were collected in 100 ms per scan. MS1 spectra were collected in the range 350–1500 m·z^− 1^, and MS2 spectra were collected in the range of 100–1500 m·z^− 1^. Precursor ions were excluded from reselection for 15 s.

### Data analysis

Protein identification and quantification were performed by a search against the UniProt *C. annuum* protein database (39,809 proteins, update in Oct. 2017). Biological modifications were selected as ID focus. Bias Correction and Background Correction was checked for protein quantification and normalization. All identified proteins had an Unused Protscore of > 1.3 (which corresponds to proteins identified with > 95% confidence), as calculated by the software and a global false discovery rate (FDR) of ≤1% determined at the protein level by the PSPEP algorithm. To be considered as being differentially expressed, proteins were required to have a *p* value of ≤0.05, as calculated by the software. For three biological replicates, the ratio of median expression between Case and Control was defined as fold changes. Statistical significance of the difference in the levels of expression of proteins between samples to be compared was determined by student’s t-test (two-tailed and unpaired) to correct for multiple hypothesis testing. For protein abundance ratios measured using iTRAQ, a 1.50-fold or 0.67-fold change threshold in the average value of three replicates with *P*-value < 0.05 in at least two experiments were classified as a significant change.

### Bioinformatics

In this study, the functional annotations of all differential abundance proteins (DAPs) were performed by using a localized Blast2go v2.6 against program against the NCBInr plant database (https://github.com/wegnerce/taxomias). The biological and functional properties of all the identified proteins were mapped with Gene Ontology (GO) Terms (http://geneontology.org/). The functional classification of the proteins using Clusters of Orthologous Groups of Proteins System (http://www.ncbi.nlm.nih.gov/COG/) was performed. All of the identified proteins were mapped to a pathway enrichment in the Kyoto Encyclopedia of Genes and Genomes (KEGG).

### Targeted protein quantification by parallel reaction monitoring (PRM)

Twelve proteins including 2 reference proteins were selected for validation by PRM on Triple TOF 5600+ LC-MS/MS system (SCIEX). Protein extraction and tryptic digestion were performed in the same way as in the iTRAQ experiment. MS data acquisition was first performed in DDA mode to obtain MS/MS spectra for the 40 most abundant precursor ions following each survey MS1 scan in each cycle. Protein Pilot software was used to identify proteins, and the database searching results were brought into Skyline software for spectra library building. Target proteins for PRM validation were imported to the software Skyline, and the peptides for protein quantification were selected according to the ion signals in spectra library. A list of associated peptides containing m/z values and retention times was exported from Skyline, and imported to MS control software Analyst for PRM acquisition method construction. PRM method was run against the biological samples of interest, evaluated and refined to develop the highest quality assay. Data collection of each sample was performed using the final PRM acquisition method on the qTOF mass spectrometer, where each precursor ion was selected by the quadrupole, fragmented, and then all fragment ions were quantified in the TOF mass analyzer. To eliminate protein carryover, a “blank” needed to be run between adjacent samples for column washing. Data processing was done in Skyline, and the quantification results were manually inspected for each peptide of the targeted proteins.

### Quantitative real-time PCR (qRT-PCR) analysis

To investigate the correlation between transcript and protein level and confirm response proteins in the iTRAQ and PRM data set, 6 different expressional proteins were selected for further qRT-PCR analysis. *Actin-97* and *phosphoenolpyruvate carboxylase 2* were set as internal reference gene. The full-length cDNA sequence of the corresponding gene was obtained from NCBI based on the interested protein information, and primers (Additional file 12: Table S9) were designed using the NCBI primer tool. After 48 h *B. tabaci* feeding, the total RNA was extracted from pepper leaves by Trizol extraction method as manufacturer’s description (Invitrogen Trading (Shanghai) Co., Ltd), and reverse transcribed cDNA from equal amounts (1.0 μg) of total RNA using the ReverTra Ace qPCR RT Kit (Toyobo life science Co.). The reactions were performed using SuperReal PreMix Plus kit (Toyobo life science Co.) according to the protocol. The thermal cycler was performed as follows: 1 cycle of 95 °C 1 min; followed by 40 cycles of 95 °C, 15 s and 60 °C, 30 s. Relative gene expression was calculated by the 2^-ΔΔCT^ method. The experiment was repeated three times.

## Additional files

Additional file 1: **Figure S1.** KEGG pathway of photosynthesis (ko00195). **PsBo,** A0A1U8FJN4/ A0A1U8FZN5; **PsBp,** A0A1U8EAE0; **PsB27,** A0A1U8FGM0; **PsaD,** A0A1U8GVK4; **PetA,** A0A1U8FRH4, **PetH,** A0A1U8FRH4; **gamma,** A0A1U8FUM0, **b,** A0A1U8GUM8 (TIF 4719 kb)

Additional file 2: **Figure S2.** KEGG pathway of carbon fixation in photosynthetic organisms (ko00710). **4.1.2.13,** A0A1U8FHQ4; **2.2.1.1,** O78327/ A0A1U8FNB3; **3.1.3.11,** A0A1U8GDS4; **2.7.2.3,** A0A1U8HDS6; **3.1.3.37,** A0A1U8GZ15; **1.1.1.40,** A0A1U8E7W8 (TIF 1471 kb)

Additional file 3: **Figure S3.** The controlled container. 50 *B. tabaci* adult insects were collected and released onto the 5th leaf and closed with a small pocket. (TIF 7155 kb)

Additional file 4: **Table S1.** Information statistics of total protein identified (XLSX 9 kb)

Additional file 5: **Table S2.** Gene ontology (GO) analysis of annotated proteins (XLSX 11 kb)

Additional file 6: **Table S3.** GO biological process enrichment DAPs (XLSX 9 kb)

Additional file 7: **Table S4.** GO molecular function enrichment of DAPs (XLSX 114 kb)

Additional file 8: **Table S5.** GO cellular component enrichment of DAPs (XLSX 46 kb)

Additional file 9: **Table S6.** Pathway enrichment of RB-RC (XLSX 17 kb)

Additional file 10: **Table S7.** Number of DAPs obtained by different analytical methods (XLSX 23 kb)

Additional file 11: **Table S8.** Parrelism between samples and iTRAQ labeling (XLSX 12 kb)

Additional file 12: **Table S9.** Primers for qRT-PCR (XLSX 12 kb)

## Data Availability

The materials used during the current study will be freely available upon request to corresponding author: fczhou@yzu.edu.cn

## Abbreviations

DAPs: differential abundance proteins
iTRAQ: isobaric tag for relative and absolute quantification
JA: jasmonic acid
PRM: parallel reaction monitoring
qRT-PCR: quantitative RT-PCR
RB: resistant genotype infested with *B. tabaci*
RC: RB control
RG: resistant genotype
SA: salicylic acid
SB: susceptible genotype infested with *B. tabaci*
SC: SB control
SG: susceptible genotype
