# Supplementary figures and images for: Proteomic analysis by iTRAQ-PRM provides integrated insight into mechanisms of resistance in pepper to Bemisia tabaci (Gennadius)
Source: BMC Plant Biol. 2019 Jun 21;19:270. doi: 10.1186/s12870-019-1849-0 (PMC6588876; doi:10.1186/s12870-019-1849-0)

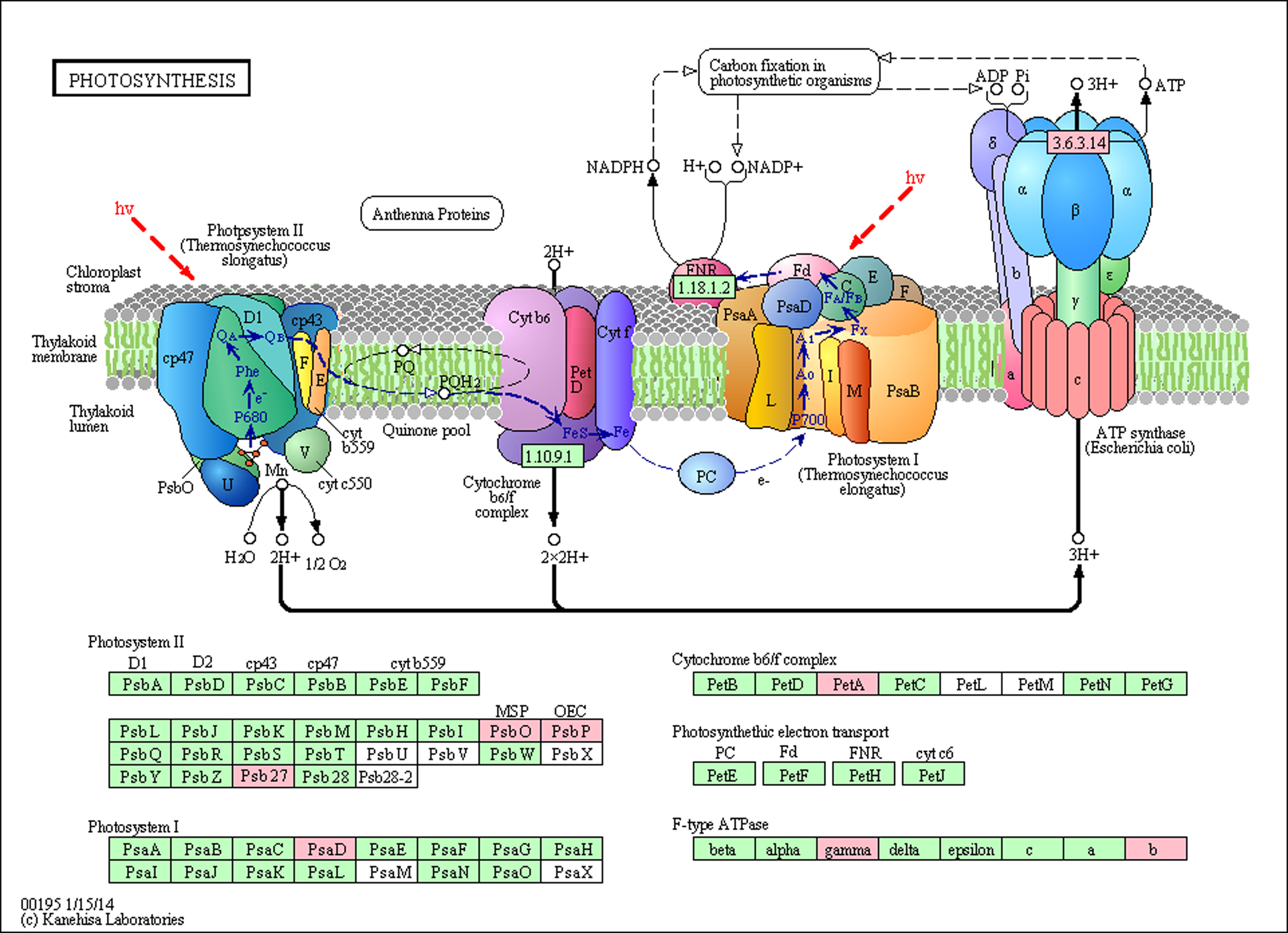

Supplement: Supplementary file 1 — Figure S1. KEGG pathway of photosynthesis (ko00195). PsBo, A0A1U8FJN4/ A0A1U8FZN5; PsBp, A0A1U8EAE0; PsB27, A0A1U8FGM0; PsaD, A0A1U8GVK4; PetA, A0A1U8FRH4, PetH, A0A1U8FRH4; gamma, A0A1U8FUM0, b, A0A1U8GUM8 (TIF 4719 kb) [file 12870_2019_1849_MOESM1_ESM.tif]

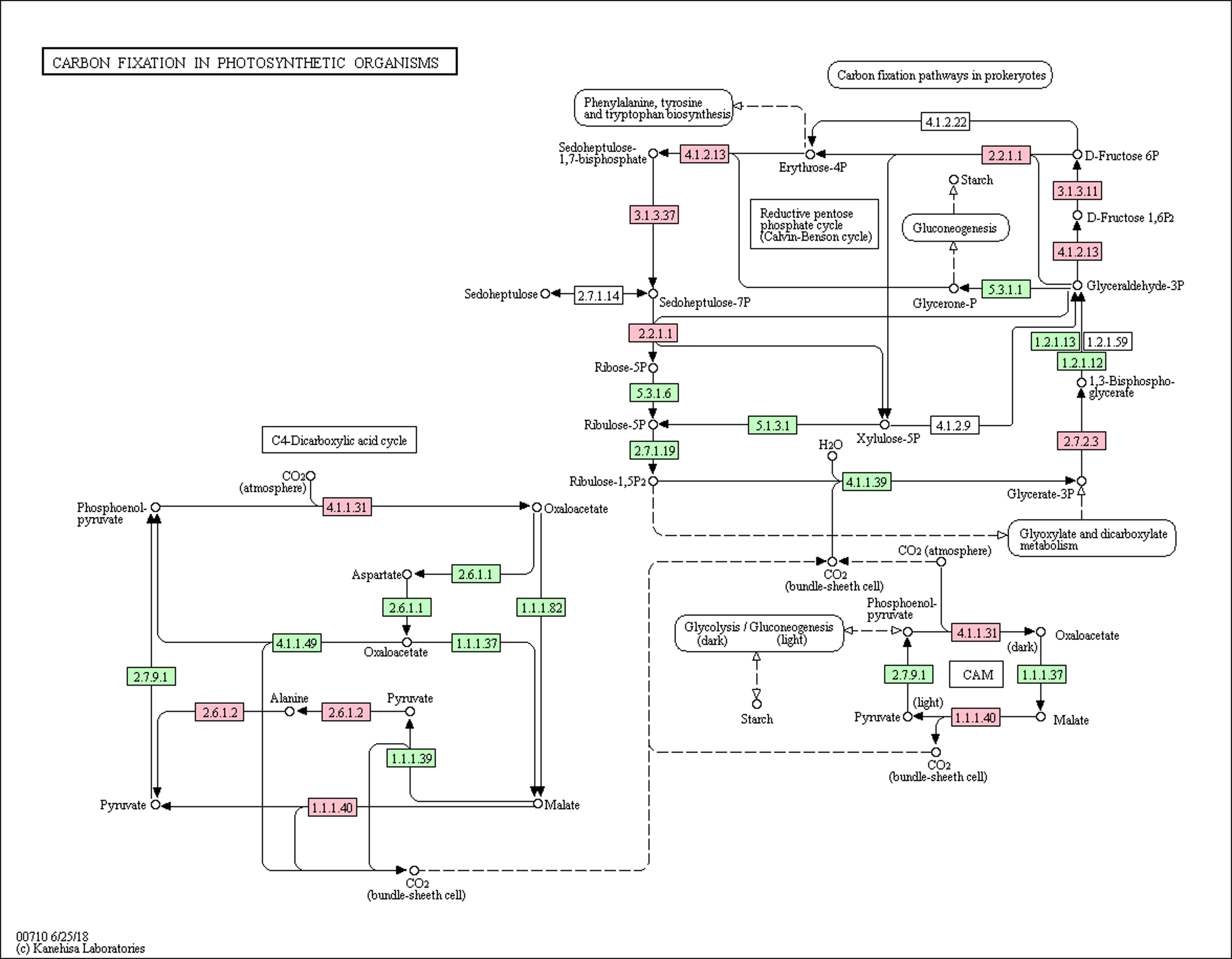

Supplement: Supplementary file 2 — Figure S2. KEGG pathway of carbon fixation in photosynthetic organisms (ko00710). 4.1.2.13, A0A1U8FHQ4; 2.2.1.1, O78327/ A0A1U8FNB3; 3.1.3.11, A0A1U8GDS4; 2.7.2.3, A0A1U8HDS6; 3.1.3.37, A0A1U8GZ15; 1.1.1.40, A0A1U8E7W8 (TIF 1471 kb) [file 12870_2019_1849_MOESM2_ESM.tif]

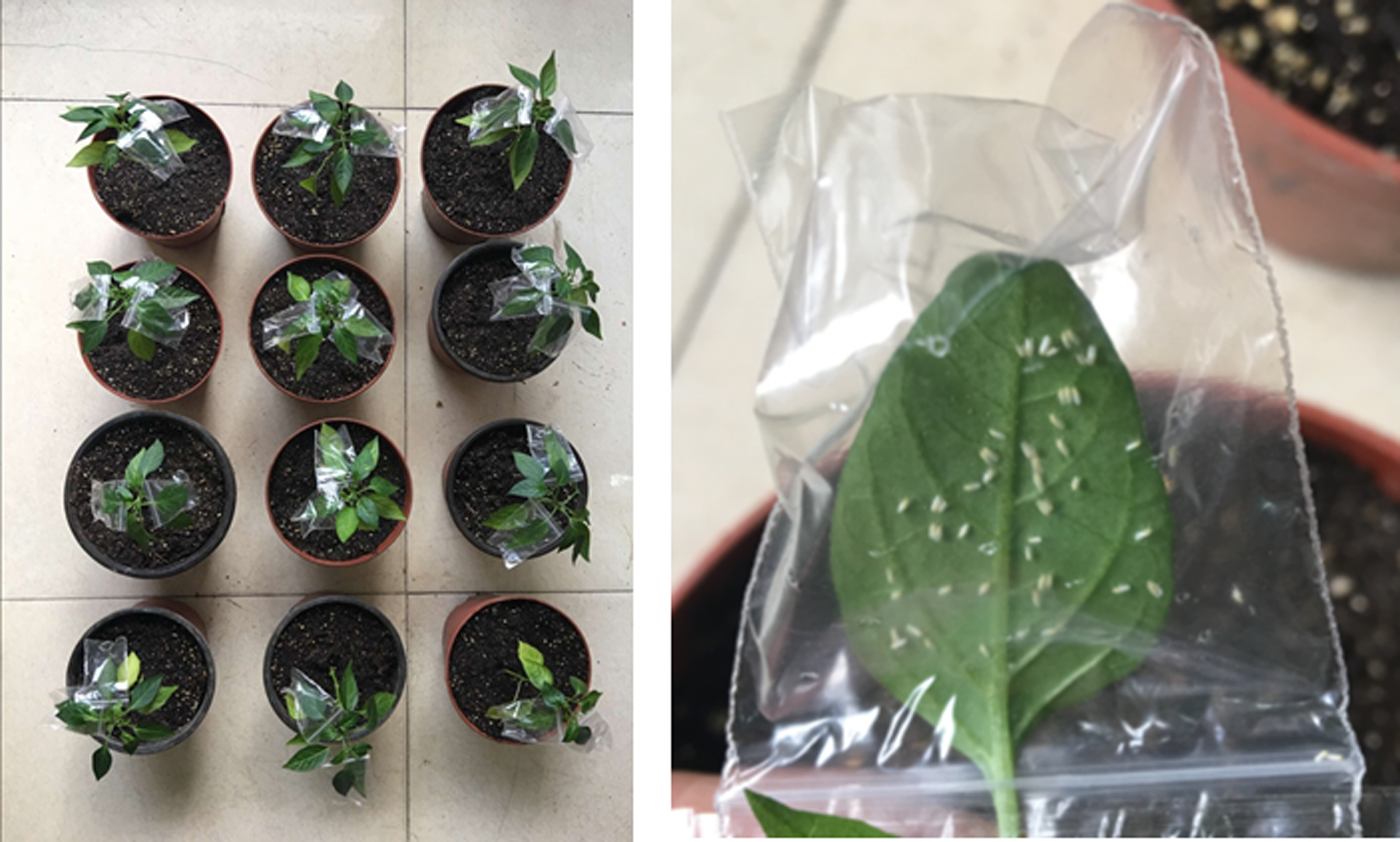

Supplement: Supplementary file 3 — Figure S3. The controlled container. 50 B. tabaci adult insects were collected and released onto the 5th leaf and closed with a small pocket. (TIF 7155 kb) [file 12870_2019_1849_MOESM3_ESM.tif]
